# An Examination of *Lactobacillus paracasei* GKS6 and *Bifidobacterium lactis* GKK2 Isolated from Infant Feces in an Aged Mouse Model

**DOI:** 10.1155/2021/6692363

**Published:** 2021-04-08

**Authors:** Shih-Wei Lin, You-Shan Tsai, Yen-Lien Chen, Ming-Fu Wang, Chin-Chu Chen, Wen-Hsin Lin, Tony J. Fang

**Affiliations:** ^1^Department of Food Science and Biotechnology, National Chung Hsing University, Taichung 402204, Taiwan; ^2^Biotech Research Institute, Grape King Bio Ltd., Taoyuan 325002, Taiwan; ^3^Department of Food and Nutrition, Providence University, Taichung 433303, Taiwan; ^4^Institute of Food Science and Technology, National Taiwan University, Taipei 106319, Taiwan; ^5^Department of Food Science, Nutrition and Nutraceutical Biotechnology, Shih Chien University, Taipei 104336, Taiwan; ^6^Department of Bioscience Technology, Chung Yuan Christian University, Taoyuan 320314, Taiwan; ^7^Department of Pharmacy, China Medical University, Taichung 404333, Taiwan

## Abstract

Supplementary which could maintain normal physiological mechanisms and functions while aging has drawn our attention due to the population aging in recent years. Probiotics have been believed with desirable properties such as antioxidation and anti-inflammatory for delaying the aging process. However, the age-related experiments conducted in the mammalian models with probiotics were few. In this study, we demonstrated the effects of administration of probiotics *Lactobacillus paracasei* GKS6 (GKS6) and *Bifidobacterium lactis* GKK2 (GKK2), respectively, at the dosage of 5.0 × 10^9^ cfu/kg BW/day for fourteen weeks in senescence-accelerated mouse prone 8 (SAMP8) mice. The three-month-old SAMP8 mice were divided into three groups: control, mice fed with GKS6, and mice fed with GKK2. There were ten females and ten males in each group. The SAMP8 mice fed with probiotics GKS6 and GKK2 showed a significantly lower degree of aging followed by Takeda's grading method on the eleventh week of the experiment. The GKK2 group showed significantly increased forelimb grip strength in male SAMP8 mice and muscle fiber number in both genders. Compared to the control, both GKS6 and GKK2 presented a significant increase in liver superoxide dismutase and catalase activities. In addition, a significant decrease in the levels of liver thiobarbituric acid-reactive substances was observed in the probiotics group. These results suggested that probiotics GKS6 and GKK2 could act as antioxidants in delaying the process of aging and preventing age-related muscle loss.

## 1. Introduction

The number and proportion of people aged sixty years and older in the population are increasing [1]. According to the report from the World Health Organization (WHO), the number of elderly (>60 years old) was calculated as one billion in 2019 and was expected to be two billion by 2050. As aging increases, the normal functions of various organs or tissues in the body gradually decline, leading to a decrease in mobility, antioxidant defense system, and immunity [2–4]. In addition, the reduction of antioxidant substances in the body would easily develop relative diseases such as immune disorder, cardiovascular diseases, and frailty syndrome [5–7]. Therefore, it is attracting attention on maintaining normal physiological mechanisms and functions during the aging process [8].

One of the age-related figures was muscle loss, including the loss of muscle mass and the loss of muscle function which could be developing to sarcopenia or dynapenia [9–11]. It has been reported that over 30% prevalence in those aged eighty years and older suffered sarcopenia [12]. Although the reason for age-related muscle decline has not been clear yet, some scientists suggested that oxidative stress, reactive oxygen species (ROS), for example, could affect cell signaling pathways, promote proteolysis, and inhibit protein synthesis in muscle fibers [13, 14]. Another explanation resulted from inflammatory cytokines IL-6 and TNF-*α*, for example, the introduction of inflammatory markers that could cause muscle breakdown, was being conducted [15, 16].

Probiotics have been reported with effects on antioxidation, anti-inflammatory, and metabolic regulation [17–19]. Although these properties were highly linked to delay the aging process, there have been few reported about antiaging in mammalian models. In our previous studies, we isolated two bacteria strains, *Lactobacillus paracasei* GKS6 (GKS6) and *Bifidobacterium lactis* GKK2 (GKK2), from healthy infant feces. Both strains presented potential properties with application as probiotics in acid and bile tolerance tests. Probiotics *L. paracasei* GKS6 had been reported with metabolic modulation in alcohol diet mouse model and antiosteoporosis in ovariectomized mice [20, 21]. *B. lactis* GKK2 showed immune enhancement in the OVA-induced murine model [22]. These evidences provided the possibility of contributing to delaying the development of aging and raised our interest [23].

The senescence-accelerated mouse prone 8 (SAMP8) is widely used in age-related studies [24]. It is a spontaneous age-accelerated mouse model of AKR/J inbred line bred by Pf. Takeda (Kyoto University, Japan) in the early 1980s [25]. The figure of SAMP8 includes comparatively old appearance, fast decline of organ function, and short life span. For the evaluation of probiotics GKS6 and GKK2 on delaying aging, SAMP8 was introduced in this study. After fourteen weeks of administration of GKS6 and GKK2, the age-associated parameters in SAMP8 were measured.

## 2. Materials and Methods

### 2.1. Bacteria Preparation

Both *Lactobacillus paracasei* GKS6 (BCRC 910788) and *Bifidobacterium lactis* GKK2 (BCRC 910826) were isolated from healthy Taiwanese infant feces. The bacteria strains were, respectively, cultured at 37°C and pH 6.0 under anaerobic conditions for 16 h with the following medium: 5% glucose, 2% yeast extract, 0.05% MgSO_4_, 0.1% K_2_HPO_4_, and 0.1% Tween-80. For harvesting, the fermented bacteria were centrifuged and mixed with protectant and then freeze-dried at 25°C for 48 hours. The live bacteria were counted by plate counting.

### 2.2. Animal Subjects

The three-month-old senescence-accelerated mice prone P8 (SAMP8) were housed under 25 ± 2°C, 65 ± 5% RH at 12 h dark/light cycle with food and water ad libitum. A total of sixty SAMP8 mice were divided into three groups (*n* = 10 in each gender): control (saline), mice fed with probiotics GKS6, and mice fed with probiotics GKK2. The probiotics were continuously given for fourteen weeks with a dosage of 5.0 × 10^9^ cfu/kg BW/day. The animal protocol in this study has been approved by the Institutional Animal Care and Use Committee (IACUC no. 20170629-A02).

### 2.3. Grading Score of Senescence

On the eleventh week of the experiment, the degree of senescence on SAMP8 was evaluated by Takeda's method [25]. The evaluation was included as follows: (1) behavior of reactivity, (2) behavior of passivity, (3) glossiness, (4) coarseness, (5) hair loss, (6) skin ulcer, (7) eye periophthalmic lesions, and (8) spine lordokyphosis. There were five grades from score 0 to score 4 representing the degree of senescence from slight to serve in each category. The total score was summed.

### 2.4. Grip Strength Test

The forelimb grip strength of SAMP8 mice was tested on the twelfth week by Grip Strength Meter (GSM 47200, Ugo Basile S.R.L., VA, Italy). The maximal force was recorded.

### 2.5. Biochemical Analysis

On the fourth week of the experiment, the SAMP8 mice blood was collected from the orbital sinus and centrifuged at 6000 rpm and at 4°C for 5 minutes and then stored at −20°C. The following parameters were analyzed by Beckman D × C 800 chemistry analyzer (Beckman Coulter, CA, USA): glucose, total protein, albumin, triglycerides, total cholesterol, high-density/low-density lipoprotein cholesterol (HDL-C/LDL-C), glutamate oxaloacetate transaminase (GOT), glutamic pyruvic transaminase (GPT), blood urea nitrogen (BUN), and creatinine.

### 2.6. Determination of Oxidative Parameters

25 mg liver tissue was homogenized with 250 *μ*l RIPA buffer and then centrifuged at 1600 ×g and 4°C for 10 minutes. The supernatant was collected and stored at −80°C for use. The superoxide dismutase (SOD) activity in the liver was analyzed by Randox assay kit (Cat. no. SD125, Randox Laboratories, ANT, UK) with absorbance at 340 nm. The catalase assay kit (Cat. no. 707002, Cayman Chemical, MI, USA) was used with 540 nm adsorption rate for the detection of catalase activity in the liver. For analyzing the levels of thiobarbituric acid reactive substances (TBARS) in mouse liver, 100 *μ*l homogenized liver supernatant was mixed with 100 *μ*l sodium dodecyl sulfate (SDS) solution and 4 ml color reagent under boiled water for an hour and then ice-cooled. The mixture was centrifuged at 1600 ×*g* under 4°C for 10 minutes and then the absorbance value was read at 535 nm spectrophotometrically. The data were expressed as equivalent malondialdehyde (MDA) *µ*M/g protein [26].

### 2.7. Immunohistochemical Analysis of Muscle Cells

After sacrifice, mouse muscle tissue was washed with 0.9% saline and fixed in 10% formalin. Tissues were embedded in paraffin and cut into 4 *μ*m thick slices for morphological and pathological evaluations. Immunohistochemical (IHC) staining of tissues involved the use of the Leica antibody to myosin heavy chain fast (WB-MHCf) and myosin heavy chain slow (WB-MHCs). By using automated BondMax with double staining, WB-MHCf and WB-MHCs epitope retrieval involved the use of ER2 (AR9640) retrieval solution for 30 min once, followed by incubation with WB-MHCf and WB-MHCs antibodies with diluent 100X for 30 min. The detection kit used was the Bond Polymer Refine Detection (DS9800) (incubation with post-primary for 8 min, polymer for 8 min, and 3′3′-diaminobenzidine for 5 min) and Bond Polymer Refine Red Detection (DS9390) (incubation with post-primary for 20 min, polymer for 30 min, red for 10 min, and haematoxylin for 5 min). Finally, results were examined under a light microscope equipped with a CCD camera (BX-51, Olympus, Tokyo) by a veterinary pathologist.

### 2.8. Statistical Analysis

Data are presented as mean ± SEM (*n* = 10 in each gender) and analyzed by one-way ANOVA with SPSS 19.0 (SPSS, NY, USA). For the comparison of statistical significance among the groups, Duncan's multiple range test was used. A *p* value <0.05 was considered statistically significant.

## 3. Results

### 3.1. Body Weight and General Characteristics of SAMP8 Mice with or without Probiotics

**Figure 1:**
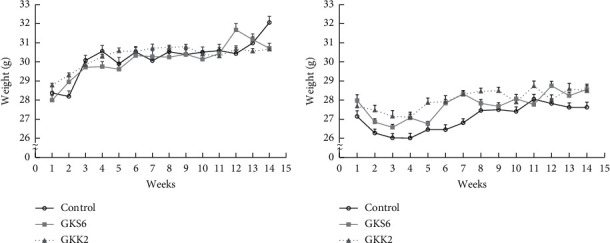
Weight changes of SAMP8 mice during the experiment. The SAMP8 male (a) and female (b) mice weights during fourteen weeks were presented as means ± SEM and analyzed by one-way ANOVA (*n* = 10). The probiotics were given at a dosage of 5.0 × 10^9^ CFU/kg BW/day. Control: SAMP8 mice fed with saline; GKS6: SAMP8 mice fed with *L. paracasei* GKS6; GKK2: SAMP8 mice fed with *B. lactis* GKK2.

There was no significant difference at the first week of all SAMP8 mice after grouping. The food intake and water consumption during the experiment were not significantly different (data not shown).  Figure 1 shows the weight changes during fourteen weeks of probiotics consumption. Male SAMP8 mice gained about two to three grams of weight on average at the end of the experiment with no significant difference among the groups (Figure 1(a)). There was also no significant difference in weight changes among the treatments in the female gender (Figure 1(b)). The relative organ weights were presented as normal in both genders when compared with the control group (Table 1). In addition, probiotics GKS6 and GKK2 did not affect biochemical parameters.

### 3.2. Effect of Probiotics *L. paracasei* GKS6 and *B. lactis* GKK2 on the Score of Senescence

**Figure 2:**
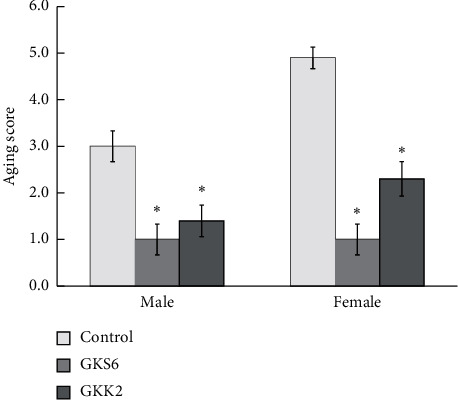
Aging score of three-month-old SAMP8 mice on the 11th week. Data were expressed as means ± SEM and analyzed by one-way ANOVA (*n* = 10). A *p* value <0.05 was regarded as a significant difference with *∗* symbol in the figure. The SAMP8 mice were given saline or probiotics at a dosage of 5.0 × 10^9^ CFU/kg BW/day. Control: SAMP8 mice fed with saline; GKS6: SAMP8 mice fed with *L. paracasei* GKS6; GKK2: SAMP8 mice fed with *B. lactis* GKK2.

The degree of senescence in three-month-old SAMP8 mice was scored on the eleventh week of the experiment. The characteristics of SAMP8 mice showed an aging appearance including dull or rough hair, hair loss, turbid eyes, and lordokyphosis. Both control male and female SAMP8 mice presented a severe senescence appearance with total grading scores at 3.0 ± 0.33 and 4.9 ± 0.23, respectively (Figure 2). Probiotics GKS6 significantly reduced the degree of senescence in skin, eyes, and spine, which presented a total aging score at 1.0 ± 0.33 in both genders. With supplementary use of probiotics GKK2, the SAMP8 male and female mice also showed a significantly lower senescence (*p* < 0.05). These results suggested that administration of probiotics GKS6 and GKK2 provided effects on delaying the process of aging.

### 3.3. Effect of Probiotics *L. paracasei* GKS6 and *B. lactis* GKK2 on Grip Strength

**Figure 3:**
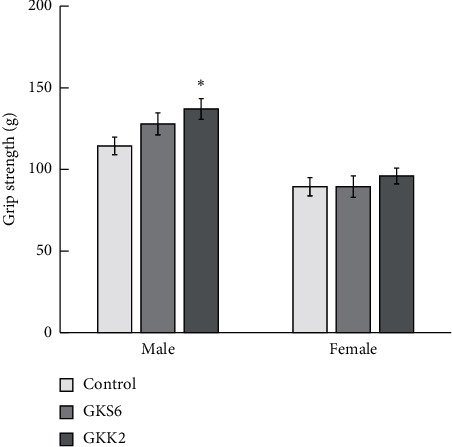
Grip strength analysis of three-month-old SAMP8 mice on the 12th week. Data were expressed as means ± SEM and analyzed by one-way ANOVA (*n* = 10). A *p* value <0.05 was regarded as a significant difference with *∗* symbol in the figure. The SAMP8 mice were given saline or probiotics at a dosage of 5.0 × 10^9^ CFU/kg BW/day. Control: SAMP8 mice fed with saline; GKS6: SAMP8 mice fed with *L. paracasei* GKS6; GKK2: SAMP8 mice fed with *B. lactis* GKK2.

In the grip strength test, control male mice presented a better grip strength than control female mice. Both probiotics GKS6 and GKK2 had no effect on the maximal peak force developed by female SAMP8 mice in a comparison with the female control (Figure 3). Administration of *L. paracasei* GKS6 showed a tendency of increased strength in male SAMP8 when compared to the male control, although it has not reached statistical significance. The male SAMP8 mice fed with *B. lactis* GKK2 presented significantly greater grip strength than the control male mice (*p* < 0.05). It is demonstrated that both probiotics GKS6 and GKK2 supplementation could alleviate the strength loss caused by aging in the male animal model.

### 3.4. Effect of Probiotics GKS6 and GKK2 on SAMP8 Mouse Muscle Cells

**Figure 4:**
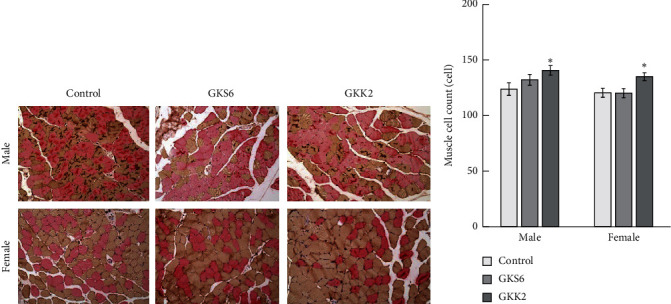
Immunohistochemical identification of muscle cell count at 200x. The muscle tissue of SAMP8 was observed by immunohistochemistry (a) and the cell count was examined (b). Type I fibers and type II fibers were stained as red and orange, respectively. The values of muscle cell count were shown as means ± S.E.M with one-way ANOVA (*n* = 10). ^*∗*^Significant difference was presented when *p* < 0.05. Control: SAMP8 mice fed with saline; GKS6: SAMP8 mice fed with *L. paracasei* GKS6; GKK2: SAMP8 mice fed with *B. lactis* GKK2.

Histological results revealed that there was no clear difference in fiber arrangement among the SAMP8 mice, as well as the proportion of type I and type II fibers (Figure 4(a)). Both GKS6 and GKK2 did not affect the fiber size in SAMP8 mice, regardless of gender. The probiotics GKS6 showed a tendency of increase in muscle cell count of male SAMP8 (Figure 4(b)). A significant increase in muscle fiber was observed in the GKK2 group when, respectively, compared to the control male and female group (*p* < 0.05). It is suggested that probiotics GKK2 could contribute to alleviating the loss of muscle fibers associated with muscle aging.

### 3.5. Effect of Probiotics GKS6 and GKK2 on Oxidative Parameters in SAMP8 Mouse

**Figure 5:**
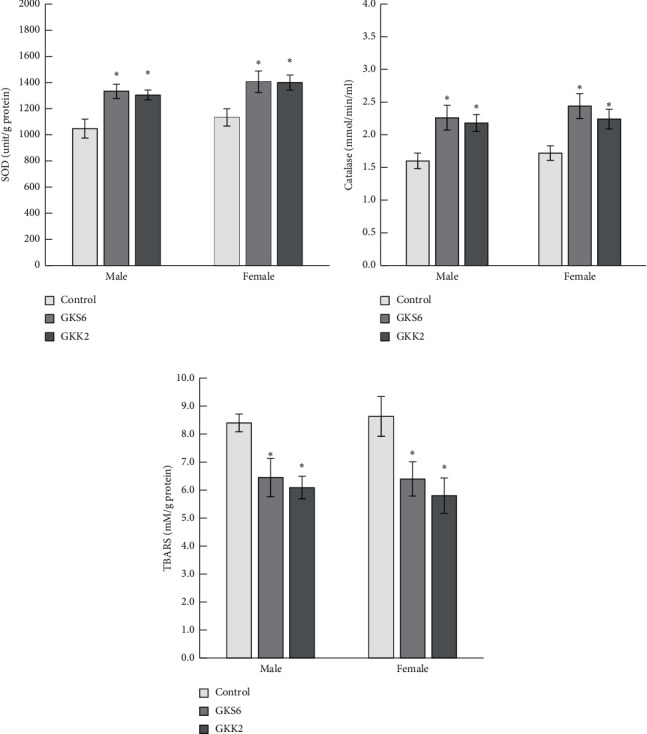
Oxidative stress parameters in the SAMP8 mouse liver. The activities of superoxide dismutase (a), catalase (b), and the concentration of thiobarbituric acid reactive substances (c) in the SAMP8 mouse liver were measured. The values were shown as means ± SEM with one-way ANOVA (*n* = 10). ^*∗*^Significant difference was presented when *p* < 0.05. Control: SAMP8 mice fed with saline; GKS6: SAMP8 mice fed with *L. paracasei* GKS6; GKK2: SAMP8 mice fed with *B. lactis* GKK2.

SOD and catalase are enzymes that involve in reactive oxygen species (ROS) scavenging. The activity of liver SOD in SAMP8 mice was significantly greater with probiotics GKS6 and GKK2, respectively, than with the control (Figure 5(a)). Similar results were observed with catalase activity in the SAMP8 mouse liver, as shown in Figure 5(b). It is indicated that both administration of GKS6 and GKK2 could improve the antioxidants activity in an aged mouse model. In addition, MDA reacted to TBARS were less detected in the liver tissue of GKS6 and GKK2 groups with *p* < 0.05 (Figure 5(c)). Furthermore, the TBARS and 8-hydoxy-2-deoxyguanosine (8-OHdG) in mouse brain were also detected with lower level in both probiotics groups when compared to the control (Figure S1). The effect of probiotics GKS6 and GKK2 on lipid oxidation during the aging process was revealed.

## 4. Discussion

In the present study, administration of probiotics GKS6 and GKK2 demonstrated a delayed effect on aging. However, the mechanisms of these two strains were supposed to be different based on our previous experiments and partial unrevealed data. There was a possibility that the effect of *B. lactis* GKK2 on antiaging was contributed by mitochondrial antioxidation; whereas the effect of *L. paracasei* GKS6 could be explained by the anti-inflammatory.

The CDGSH iron-sulfur domain 2 (Cisd2), a redox-sensitive gene, was reported with a crucial role in the lifespan and the development of age-related diseases [27]. In our preliminary test, an enhancement of Cisd2 gene expression in HEK293T cells was presented in GKK2 treatment but not in the GKS6 group (Figure S2). A persistent level of Cisd2 protected mitochondrial dysregulation and reduced DNA damage caused by oxidative stress; in addition, Cisd2 was involved in calcium homeostasis through the regulation of the calcium channels located on the endoplasmic reticulum and mitochondrial outer membranes [28–30]. These affections maintain a better physical function in skeletal muscle, liver, and heart [31, 32]. The Cisd2 mKO mice showed a similar pattern as naturally aged mice in the decline of gastrocnemius muscle [33]. It explained that GKK2 increased grip strength and muscle mass in aged-accelerated mouse models involved with the regulation of the cisd2 gene.

Although GKS6 got lower aging scores than GKK2 in the grading system (Figure 2), it seems like age-dependent muscular parameters were not affected a lot by GKS6 than GKK2. Therefore, it gave us clues that probiotics affect differently on antiaging from strain to strain. We also investigated the ratio of bone volume/tissue volume (BV/TV), trabecular thickness (Tb. Th), trabecular number (Tb. N), trabecular separation (Tb. Sp), and bone mineral density (BMD) in female SAMP8 mice fed with GKS6 (Table S1). Even though the results did not reach statistical significance, the trends in the decrease of Tb. Sp and increase of BMD were similar to our previous study in an ovariectomized mice model [21]. Interestingly, GKS6 relatively maintained higher BV/TV (%) and Tb. N (No./mm) in SAMP8 females which was not observed in the past. Generally, bone loss due to aging is regarded as a chronic inflammatory state involving increased proinflammatory cytokines such as IL-6, IL-1, and receptor activator of nuclear factor-*κ*B ligand (RANKL) [34, 35]. According to the inhibited effect of GKS6 with RANKL treatment on RANK, the functions of GKS6 in this study on age-related bone loss via anti-inflammatory pathway could be surmised [21].

It could be expected that the combination of GKS6 and GKK2 has a potential synergistic effect on improving age-related symptoms since they might work differently in physiological function. Fu et al. revealed a similar outcome of antiaging effect with oral *Lactobacillus* spp. and *Bifidobacterium* spp., respectively, in C57BL/6 mice; however, the compositions of gut microbiota in mice were very different between these two treatments [36]. The different role of probiotics on antiaging could provide the complexity of physiological modulation which builds a multiple defense system, that is, providing more appropriate solutions and less bad effect concern. In this study, we contributed to provide two different probiotic strains with different mechanisms for antiaging effect.

## 5. Conclusions

In this study, we demonstrated that dietary supplementation of *L. paracasei* GKS6 and *B. lactis* GKK2 in SAMP8 mice was safe. In addition, administration of probiotics GKS6 and GKK2 significantly delayed the aging process by enhancing antioxidants activity, resulting in lower oxidative damage. Moreover, *B. lactis* GKK2 showed a significant effect on forelimb strength strengthening and muscle fiber hypergenesis. Both *L*. *paracasei* GKS6 and *B. lactis* GKK2 could act as candidates of functional food for antiaging. *B. lactis* GKK2 could further be a potential supplementary as an elderly muscle-building diet.

## Figures and Tables

**Table 1:** Relative organ weights and plasma biochemical parameters of SAMP8 mice.

	Male			Female		
Group	Control	GKS6	GKK2	Control	GKS6	GKK2
Relative weight (g/100 g body weight)						
Brain	1.440 ± 0.038^a^	1.535 ± 0.012^a^	1.524 ± 0.017^a^	1.736 ± 0.043^a^	1.782 ± 0.049^a^	1.769 ± 0.061^a^
Heart	0.662 ± 0.025^a^	0.651 ± 0.023^a^	0.673 ± 0.018^a^	0.564 ± 0.017^a^	0.597 ± 0.025^a^	0.556 ± 0.026^a^
Liver	4.853 ± 0.282^a^	4.829 ± 0.235^a^	4.369 ± 0.174^a^	4.651 ± 0.201^a^	4.442 ± 0.115^a^	4.768 ± 0.288^a^
Spleen	0.352 ± 0.037^a^	0.294 ± 0.015^a^	0.264 ± 0.018^a^	0.431 ± 0.037^a^	0.444 ± 0.023^a^	0.458 ± 0.033^a^
Lung	0.720 ± 0.030^a^	0.805 ± 0.033^a^	0.798 ± 0.045^a^	0.836 ± 0.045^a^	0.772 ± 0.020^a^	0.860 ± 0.033^a^
Kidney	1.710 ± 0.064^a^	1.713 ± 0.046^a^	1.663 ± 0.074^a^	1.264 ± 0.020^a^	1.264 ± 0.021^a^	1.273 ± 0.044^a^
Plasma biochemical parameters						
Albumin (g/d)	2.92 ± 0.09^a^	3.11 ± 0.07^a^	3.14 ± 0.06^a^	3.45 ± 0.07^a^	3.50 ± 0.09^a^	3.50 ± 0.11^a^
Glucose (mg/dl)	154.5 ± 5.47^a^	159.90 ± 4.14^a^	159.10 ± 5.34^a^	162.60 ± 5.66^a^	155.30 ± 5.81^a^	161.10 ± 3.57^a^
Total cholesterol (mg/dl)	138.10 ± 6.81^a^	145.30 ± 4.71^a^	144.20 ± 4.58^a^	99.50 ± 2.98^a^	100.90 ± 2.18^a^	101.90 ± 2.61^a^
Triglyceride (mg/dl)	97.30 ± 3.36^a^	99.30 ± 3.16^a^	101.30 ± 4.35^a^	98.90 ± 3.05^a^	100.60 ± 4.11^a^	99.60 ± 1.10^a^
HDL (mg/dl)	109.81 ± 5.14^a^	108.73 ± 2.71^a^	112.84 ± 4.58^a^	61.01 ± 4.88^a^	53.57 ± 5.70^a^	46.25 ± 6.12^a^
Total protein (mg/dl)	5.94 ± 0.15^a^	6.32 ± 0.22^a^	6.44 ± 0.22^a^	6.06 ± 0.10^a^	6.04 ± 0.07^a^	5.89 ± 0.11^a^
LDL (mg/dl)	18.70 ± 2.65^a^	18.20 ± 1.73^a^	22.10 ± 1.42^a^	9.17 ± 0.96^a^	7.88 ± 0.10^a^	18.87 ± 5.73^a^
GPT (U/L)	47.40 ± 5.53^a^	45.80 ± 3.85^a^	46.30 ± 3.80^a^	61.30 ± 5.36^a^	63.70 ± 5.43^a^	64.10 ± 4.47^a^
GOT (U/L)	181.80 ± 17.01^a^	179.20 ± 9.53^a^	173.40 ± 10.99^a^	178.80 ± 9.40^a^	172.70 ± 11.22^a^	177.70 ± 12.70^a^
BUN (mg/dl)	27.19 ± 1.96^a^	26.95 ± 0.73^a^	29.27 ± 2.34^a^	25.93 ± 2.00^a^	23.54 ± 0.72^a^	25.78 ± 2.21^a^
Creatinine (mg/dl)	0.31 ± 0.02^a^	0.33 ± 0.02^a^	0.30 ± 0.22^a^	0.32 ± 0.02^a^	0.35 ± 0.01^a^	0.37 ± 0.02^a^

Values were presented as mean ± SEM (*n* = 10) (one-way ANOVA). Alphabet “a” represented no significant difference by Duncan's multiple range test. The SAMP8 mice were given saline or probiotics at a dosage of 5.0 × 10^9^ CFU/kg BW/day. GKS6 : *L. paracasei* GKS6; GKK2 : *B. lactis* GKK2.

## Data Availability

All the data used to support the findings of this study are included within the article.

## Supplementary Materials

Supplementary Materials Figure S1: oxidative stress parameters in SAMP8 mouse brain. The concentrations of thiobarbituric acid reactive substances (a) and 8-hydoxy-2-deoxyguanosine (b) in SAMP8 mouse brain were measured. The values were shown as means ± SEM with one-way ANOVA (*n* = 10). ^*∗*^Significant difference was presented when *p* < 0.05. Control: SAMP8 mice fed with saline; GKS6: SAMP8 mice fed with *L. paracasei* GKS6; GKK2: SAMP8 mice fed with *B. lactis* GKK2. Figure S2: Cisd2 expression with probiotic treatments on HEK 293T cell line. A screening test from human embryonic kidney 293T (HEK 293T) cell line revealed by western blot (Fig. S2a) and the Cisd2 expression was determined as a relative percentage of the control (Fig. S2b). GKS6: *L. paracasei* GKS6; GKK2: *B. lactis* GKK2; Cisd2: CDGSH iron-sulfur domain 2; GAPDH: glyceraldehyde-3-phosphate dehydrogenase. Table S1: effect of probiotics on bone parameters in SAMP8 female mice.
