# Supplementary material for: An Examination of Lactobacillus paracasei GKS6 and Bifidobacterium lactis GKK2 Isolated from Infant Feces in an Aged Mouse Model
Source: Evid Based Complement Alternat Med. 2021 Apr 8;2021:6692363. doi: 10.1155/2021/6692363 (PMC8052140; doi:10.1155/2021/6692363)
Supplement: Supplementary Materials — Figure S1: oxidative stress parameters in SAMP8 mouse brain. The concentrations of thiobarbituric acid reactive substances (a) and 8-hydoxy-2-deoxyguanosine (b) in SAMP8 mouse brain were measured. The values were shown as means ± SEM with one-way ANOVA (n = 10). ∗Significant difference was presented when p < 0.05. Control: SAMP8 mice fed with saline; GKS6: SAMP8 mice fed with L. paracasei GKS6; GKK2: SAMP8 mice fed with B. lactis GKK2. Figure S2: Cisd2 expression with probiotic treatments on HEK 293T cell line. A screening test from human embryonic kidney 293T (HEK 293T) cell line revealed by western blot (Fig. S2a) and the Cisd2 expression was determined as a relative percentage of the control (Fig. S2b). GKS6: L. paracasei GKS6; GKK2: B. lactis GKK2; Cisd2: CDGSH iron-sulfur domain 2; GAPDH: glyceraldehyde-3-phosphate dehydrogenase. Table S1: effect of probiotics on bone parameters in SAMP8 female mice. [file 6692363.f1.docx]

## **Supplementary Materials**


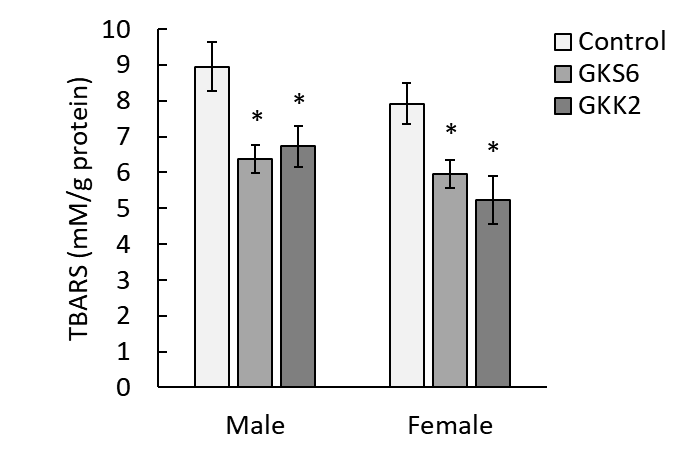

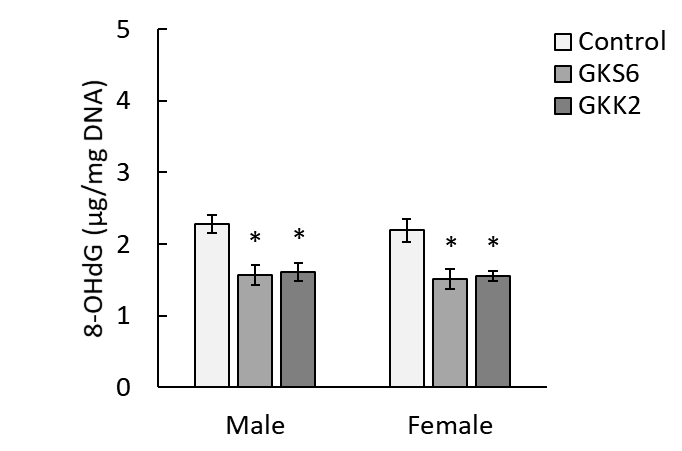


(a)

(b)

**Figure S1: Oxidative stress parameters in SAMP8 mouse brain.** The concentration of thiobarbituric acid reactive substances (a) and 8-hydoxy-2-deoxyguanosine (b) in SAMP8 mouse brain were measured. The values were showed as means ± S.E.M with one-way ANOVA analysis (n=10). * significant difference was presented when *p*<0.05. Control: SAMP8 mice fed with saline; GKS6: SAMP8 mice fed with *L. paracasei* GKS6; GKK2: SAMP8 mice fed with *B. lactis* GKK2.


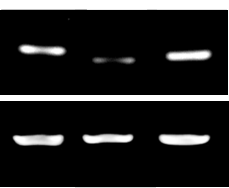

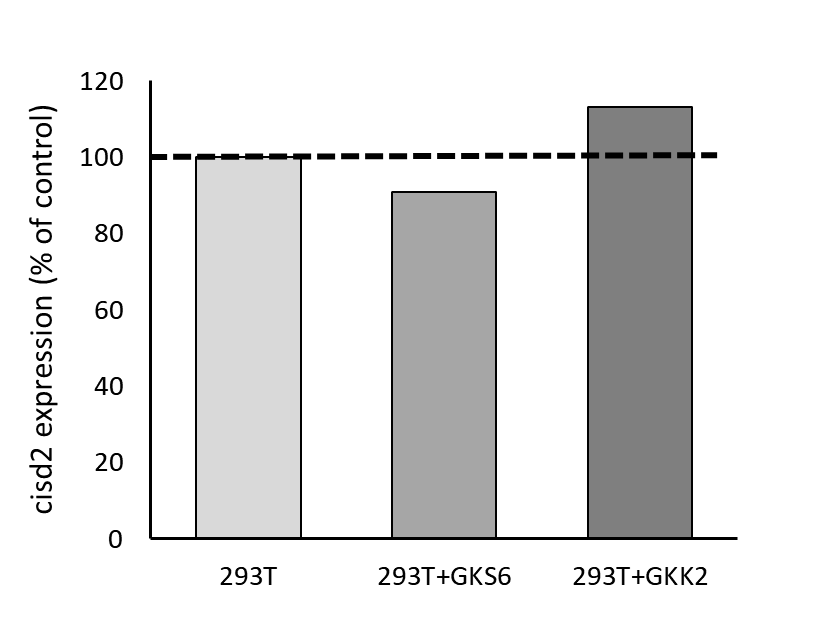


Cisd2

GAPDH

Ctrl

GKS6

GKK2

(a)

(b)

**Figure S2: Cisd2 expression with probiotic treatments on HEK 293T cell line.** A screening test from human embryonic kidney 293T (HEK 293T) cell line revealed by western blot (Fig. S2a) and the cisd2 expression was determined as relative percentage of the control (Fig. S2b). GKS6: *L. paracasei* GKS6; GKK2: *B. lactis* GKK2; Cisd2: CDGSH iron sulfur domain 2; GAPDH: glyceraldehyde-3-phosphate dehydrogenase.

**Table S1: Effect of probiotics on bone parameters in SAMP8 female mice.**

| Treatment |  | Control |  | GKS6 | Trend |  | GKK2 | Trend |
| --- | --- | --- | --- | --- | --- | --- | --- | --- |
| BV/TV (%) |  | 3.18±0.14 |  | 3.61±0.13 | ↑* |  | 3.40±0.11 | - |
| Tb. Th (μm) |  | 0.07±0.00 |  | 0.08±0.00 | - |  | 0.07±0.00 | - |
| Tb. N (No./mm) |  | 0.40±0.02 |  | 0.47±0.03 | ↑* |  | 0.44±0.02 | ↑ |
| Tb. Sp (μm) |  | 0.49±0.02 |  | 0.46±0.02 | ↓ |  | 0.48±0.02 | - |
| BMD (g/cm^3^) |  | 0.42±0.02 |  | 0.46±0.02 | ↑ |  | 0.42±0.02 | - |

Values were presented as mean ± S.E.M., n=10. Symbol ↑ and ↓represented the increased and decreased trend respectively with a comparison of the control. *A statistical significance was marked. GKS6: *L. paracasei* GKS6; GKK2: *B. lactis* GKK2.
